# A telephone- and text-message based telemedical care concept for patients with mental health disorders - study protocol for a randomized, controlled study design

**DOI:** 10.1186/1471-244X-11-30

**Published:** 2011-02-17

**Authors:** Neeltje van den Berg, Hans-Jörgen Grabe, Harald J Freyberger, Wolfgang Hoffmann

**Affiliations:** 1 Institute for Community Medicine University of Greifswald Ellernholzstr. 1/2 17487 Greifswald, Germany; 2 Department of Psychiatry and Psychotherapy University of Greifswald Ellernholzstr. 1/2 17487 Greifswald, Germany

## Abstract

**Background:**

As in other countries worldwide, the prevalence of mental disorders in Germany is high. Although numerically a dense network of in- and outpatient psychiatric health services exists, the availability in rural and remote regions is insufficient.

In rural regions, telemedical concepts can be a chance to unburden and complement the existing healthcare system. Telemedical concepts consisting of video or telephone consulting show first positive results, but there are only a few studies with a randomized controlled design.

To improve the treatment of patients with mental disorders in rural regions, we developed a telemedical care concept based on telephone contacts and text-messages. The primary objective of this study is to evaluate the effects of the telemedical interventions on psychopathological outcomes, e. g. anxiety, depressive symptoms, and somatisation. Secondary objective of the study is the analysis of intervention effects on the frequency of medical contacts with healthcare services. Furthermore, the frequency of patients' crises and the frequency and kind of interventions, initiated by the project nurses will be evaluated. We will also evaluate the acceptance of the telemedical care concept by the patients.

**Methods/Design:**

In this paper we describe a three-armed, randomized, controlled study. All participants are recruited from psychiatric day hospitals. The inclusion criteria are a specialist-diagnosed depression, anxiety disorder, adjustment disorder or a somatoform disorder and eligibility to participate in the study. Exclusion criteria are ongoing outpatient psychotherapy, planned interval treatment at the day clinic and expected recurrent suicidality and self-injuring behaviour.

The interventions consist of regular patient-individual telephone consultations or telephone consultations with complementing text-messages on the patients' mobile phone. The interventions will be conducted during a time period of 6 months.

**Trial registration:**

This study is registered in the German Clinical Trials Register (DRKS00000662).

## Background

### Epidemiology and utilization of health care services

Surveys and cohort studies have shown that mental disorders have high prevalence rates in the general population worldwide. For example the representative National Comorbidity Survey Replication in the USA with over 9 000 participants showed a lifetime prevalence of 46.4% for any mental disorder, thereof 28.8% for anxiety disorders and 20.8% for mood disorders [1].

In Germany, the German Health Interview and Examination Survey (GHS) revealed similar results. A lifetime prevalence of 43% for any disorder was found, thereof the most frequent mental diseases were anxiety, mood, and somatoform disorders [2]. The German Federal Health Survey of 1998 (Bundes-Gesundheitssurvey) showed a lifetime prevalence of 19% for depression [3]. The German findings are comparable to those in other European countries [4].

In Germany, health care in the field of mental diseases consists of psychotherapeutic practices for outpatient care, specialised clinics or departments in hospitals for inpatient treatment, and also day care hospitals, psychiatric walk-in clinics, and other psychiatric information and consultation centres are established [5].

However, regarding the availability of outpatient psychotherapists, there are large regional differences. The number of inhabitants per psychotherapist fluctuates between 2577 in cities and 23106 in rural regions [5]. Consequences are long waiting lists, e.g. for a continuation of treatment after delivery from a psychiatric hospital or day hospital.

As shown in several studies, treatment rates are fairly low. In their review of 27 studies, covering 16 European countries, Wittchen and Jacobi found that only 26% of all patients had any contact with health care services [4]. In Germany, the German Health Interview and Examination Survey showed health care utilization of 30% for patients without co-morbidity and 76% for highly co-morbid patients [2]. In the cohort "Study of Health in Pomerania" (SHIP) [6], which is conducted in the study region Western Pomerania, a treatment rate of 20% was estimated [7].

Mental disorders are associated with increased usage of healthcare services [8]. In the SHIP-cohort, depression and somatisation and a combination of depression, somatisation, and anxiety were predictors for considerable increases of inpatient and outpatient costs between baseline and 5-year follow-up [9].

### Telemedical concepts for mental healthcare

To improve the treatment of patients with mental disorders, especially in rural regions, telemedical concepts can be a chance to unburden and complement the existing healthcare system.

In the last years, several studies to evaluate possibilities and limitations of telemedical concepts were applied in various countries. In some cases, videoconferences were conducted to enable contacts with psychotherapists. The results of a project in Canada, that evaluated the satisfaction of healthcare professionals with video consultations between patients in rural areas and general hospitals, were positive [10]. In a project on the Canary Islands, videoconferencing was established to provide psychiatric consultations for remote regions. Patients' acceptance and satisfaction with the concept were high. Video consultation was used mostly to confirm diagnoses of the local general practitioners and to get advice about case management of the patients [11]. In Australia, the feasibility of video consultations in child and adolescent psychiatry was evaluated. The results were positive, video consultations were seen as a flexible and effective service for patients with complex needs [12].

A randomized study from Ontario, Canada, analysed clinical outcomes of patients, who received telepsychiatric consultations compared to patients receiving face-to-face consultations. The clinical outcomes of both groups were equivalent [13]. Reviews of randomized controlled trials show, that videoconferencing can be a good alternative for face-to-face consultations, especially in rural regions. The acceptance among patients and psychotherapists is high, clinical outcomes are comparable with usual care [14-16].

Video consultations require a high level of organisation, special technical conditions, and financial investment. Telephone consultations are, technical, organisational, and financial, less demanding but maybe perceived as more impersonal as video consultations. An advantage is the broad and permanent availability of mobile phones, which can be beneficial in crisis situations.

Hilty et al. describe a concept of using telephone consultation and e-mail contacts. The participating patients showed clinical improvement, the providers were satisfied with the concept [17].

Another (non-randomized) study (cognitive behavioral therapy-telephone treatment (CBT-TT)) evaluated telephone-psychotherapy for patients with depression, which initiated a treatment at a mental health clinic. After 3 and 6 months, there was a significant reduction in depression severity [18].

In a randomized controlled trial, among others, telephone therapy was compared with usual care with patients with depressive disorders, starting their therapy. The telephone psychotherapy intervention resulted in a significant reduction of depression severity [19].

Rollman et al. examined whether telephone-based collaborative care can improve clinical outcomes for panic and generalized anxiety disorders compared to usual care provided by primary care physicians. After 12 months, both anxiety and depressive symptoms improved [20].

A randomized controlled trial in Germany, conducted in GP-practices, found a significant decrease of the severity of depression symptoms. In this trial, structured telephone interviews to assess depression symptoms were made by practice assistants [21].

Although Germany has large rural regions with an insufficient availability of outpatient psychotherapeutic healthcare services, the awareness of the potentially beneficial contribution of telemedical consultations in this field is still poorly developed.

The study outlined in this paper is based on collaboration between the Clinic of Psychiatry and Psychotherapy and the Institute for Community Medicine, both located at the University of Greifswald in the region of Western Pomerania in Germany. A telemedical centre is affiliated with the Institute for Community Medicine. Here, telemedical concepts for various indications and patients groups are developed, implemented, and evaluated [22].

Western Pomerania is a rural region in the Northeast of Germany at the Baltic Sea coast. Psychotherapeutic healthcare services are concentrated in the larger towns. After treatment in a psychiatric day hospital, patients have to wait up to 6 months for further treatment in a regular outpatient psychotherapeutic practice.

To bridge this long waiting period, we developed a telemedical concept consisting of regular, patient-centred telephone consultations and text-messages on the mobile phone, conducted by nurses of the University Hospital of Greifswald.

The intention of this concept is to ensure a low-threshold continuous telemedical care beyond discharge from a psychiatric day hospital, to attend to patients` crises timely and to initiate necessary interventions.

## Research objectives

The primary objective of this study is to evaluate the effects of the telemedical interventions on psychopathological outcomes, e. g. anxiety, depressive symptoms, and somatisation.

Secondary objective of the study is the analysis of effects of the interventions on the frequency of medical contacts with healthcare services, both psychotherapeutic and in other medical fields.

Furthermore, we will evaluate the frequency of patients' crises, recognized by the project nurses, the frequency and kind of interventions, initiated by the project nurses, and also assess the kind of medication during the intervention.

We will also evaluate the acceptance of the telemedical care concept by the patients.

## Methods/Design

### Study design

This study is a three-armed, prospective, controlled, randomized trial. Two of the study arms include an intervention (regular telephone contacts and telephone contacts with additional text-messages), the third arm is a control group. The interventions will be conducted during a time period of 6 months after discharge. Importantly, the interventions outlined below are all applied in addition to the individual outpatient treatment provided. Therefore, also the control group receives outpatient treatment (e.g. medication, short interventions by any GP or psychiatrist).

The outcomes described in the section research objectives will be compared between all three groups.

### Recruiting and participants

It was planned to recruit a total of 90 patients, 30 in each arm of the study. The recruitment started September 2009. As the first participants were contacted to have their final interview after 6 months, the loss to follow up in the control group was about 20%. Therefore, we decided to continue the recruitment until a total of 120 participants included.

All participants are recruited from three psychiatric day hospitals in the region of Western Pomerania in the Northeast of Germany. The treating psychiatrists and psychotherapists select eligible patients before their discharge from the day hospital. The patients are informed about the project and are asked to provide informed consent.

The inclusion criteria are:

- a diagnosed depression, anxiety disorder, adjustment disorder or a somatoform disorder;

- eligibility to participate in the study, attested by the treating psychiatrist or psychotherapist.

Exclusion criteria are

- interval patients, defined as patients who return to the day hospital after 3-6 months to continue their therapy;

- patients who show a distinct emotional instability with recurrent suicide crises and self-injuring behaviour.

After the patients' agreement to participate in the study, the treating psychiatrist or psychotherapist from the day clinic completes a short standardized enrolment form with personal data of the patient (name, address, telephone number, date of birth), diagnoses, medication, and patients' individual therapy goals and/or themes, that were elaborated between the psychotherapist and the patient. Examples for therapy goals or themes are: exposure to critical factors (e.g. to use public transportation, to go shopping alone), to socialize with other people, to deal with family problems, to perform relaxation techniques regularly, to create a structural schedule for the day and the week, to concern about occupational rehabilitation. The enrolment form is transferred to the telemedical centre. Here, the patients are randomized to one of the three study arms.

### Study intervention

Two telemedical interventions of different intensity will be applied during a time period of 6 months. The intervention of the first arm of the study consists of regular telephone contacts, conducted by specially trained nurses. The first month, the telephone contacts take place once a week, thereafter, once a month. If necessary, the frequency of the telephone contacts can be increased. It is also possible for the patients to contact the nurses by telephone during office hours (8 am - 4 pm).

The first part of the telephone calls consists of standardized questionnaires to enable the monitoring of important parameters over time:

- The Brief Symptom Inventory (BSI) is a standardized questionnaire to assess the severity of relevant symptoms, e.g. feelings of loneliness, melancholia, panic attacks, restlessness, suicidal thoughts, pain in heart and chest, sickness [23].

- Contacts with physicians: assessment of the number of contacts with a general practitioner, an emergency physician, and various medical specialists and the reason for the last physician contact. Further, the patient is asked to evaluate his satisfaction with the contacts applying grades from 1 to 6.

- Inpatient stays: it is assessed whether the participants had inpatient stays in hospitals (separate for acute and planned admission), and rehab centres. The participants are asked for the number of stays, the total number of days, and the reason for admission. During the first telephone call, contacts with physicians and inpatients stays are assessed for the last 6 months, during the following contacts for the time since the last telephone contact.

- Standardized evaluation of the health situation of the participant by the nurse, it is possible to supplement this judgement by free text remarks.

The second part of the telephone call consists of asking for special or unusual occurrences and specific questions about the individual therapy themes as a guideline for the talk:

- Did anything special or unusual happen during the last weeks (e.g. regarding family, relationship, friends, job, health situation)? Was it positive or negative? Were you satisfied with your behaviour in or reaction on this situation?

- For each therapy goal or theme: in the day hospital, you formulated the following therapy goal or theme together with your psychotherapist. Could you work on it? Are you satisfied about how you worked on it (grade 1 to 6)? Do you think you can pursue this goal more intensively?

The third part of the telephone call deals with the medication of the participant. The following questions are asked for each drug separately:

- Do you take this drug in the same dosage as the last time we called (first telephone call: as you were discharged from the day hospital)? If no, why not? Did a physician change something about the dosage? If yes, what was changed?

- How do you assess the effect of this drug (free text)?

- How regular do you take this drug? Possible answers: always, mostly, rarely, never, don't know, don't want to answer

The telephone call is finalized by making an agreement about the next call and the question whether the patient wishes to include another topic.

The intervention of the second study arm consists of telephone contacts with the same content as the telephone contacts of the first study arm as described above. Additionally, once a week short text-messages are sent to the participating patients. These text-messages take up the individual therapy goals or themes (e.g. "Did you take the bus today?", Did your appointment with your boss go well?"). The participants can answer on these messages, and the nurses will react again if necessary or appropriate.

If the nurses recognize a (starting) crisis, they will increase the number of telephone contacts, make an appointment for a consultation with the treating psychotherapist or arrange a crisis intervention by the treating psychotherapist or by the hospital.

### Evaluation Interviews

For the patients in the intervention arms of the study, evaluation data is collected mainly during the standardized part of the telephone calls (sociodemographic parameters, Brief Symptom Inventory, contacts with health services, medication assessment). Additionally, there is a short interview about acceptance of and satisfaction with the telemedical care concept [Table 1].

**Table 1:** Interview questions to assess acceptance and satisfaction of the patients

Question:	How do you judge the telephone contacts during the last 6 momths?
Answers:	Very helpful - helpful - not helpful - other (free text) - I don't know - I don't want to answer
Question:	Would you be interested to continue the telephone contacts if possible?
Answers:	Yes - No - I don't know - I don't want to answer
Question:	Do you think, this kind of care can make face-to-face contacts less necessary or replace them partly?
Answers:	Yes - No - I don't know - I don't want to answer
Question:	Is there something you would change or improve?
Answer:	Free text

The patients of the control group have a baseline interview and a follow up interview after 6 months which includes the same standardized questionnaires as the intervention groups.

**Figure 1:**
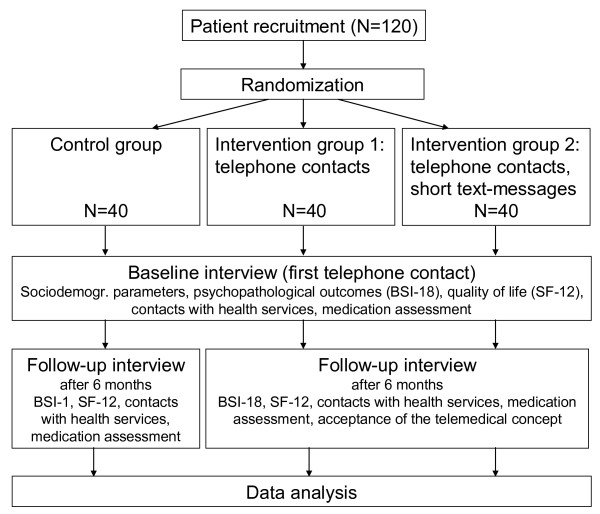
Flow chart of the study

A flow chart of the study is shown in Figure 1.

### Documentation, data storage and data security

Most of the data are documented during the telephone calls supported by a special developed computer aided documentation system.

Personal data of the patients, diagnoses, the first assessment of the medication, and the therapy themes are abstracted from the enrolment form and transferred to the project database. For the evaluation of contacts with health services, we will also apply for data from hospital-IT-systems of various hospitals within the region of Western Pomerania and the patients' statutory health insurances.

All data are stored in a central data management system, based on a mySQL-database. The system is built following actual standards for data security and availability. Since the system is mirrored, data collection, storage, and availability are not endangered in case of problems with the central system. Data security and availability are ensured anytime [24].

### Data protection

All participating patients provide written informed consent after detailed information by the treating psychotherapist in the day hospital. The original forms are stored in a closed cabinet, the patients receive a copy.

If a patient withdraws his consent partly or fully, this is noted in the documentation and no new data (to the extent the patient defined) will be included. The patient's data will not be used in the analyses and, if this is the patient's wish, will be deleted from the project database. Only those data that have already used in analyses and project results are excluded.

Within the project database, identifying data is stored separately from the collected data. Only project staff with specifically conferred access rights has access to the identifying data.

After finalizing the data collection phase of the study, all the identifying variables will be physically separated from the other data. Data analysis will be conducted in a strictly pseudonymised way.

### Ethics approval

The study is conducted in compliance with ethical requirements as testified by the institutional ethics committee of the board of physicians Mecklenburg-Western Pomerania at the University of Greifswald (approval at 2009\06\30, reg. nr. BB 50/09)

### Trial Registration

This study is registered in the German Clinical Trials Register (DRKS00000662).

## Analysis

After finalizing the recruiting of the participating patients and conducting the interventions, the collected data will be analysed in a strictly pseudonymised way. Three kinds of analyses will be applied:

- The clinical outcomes of the patients (e. g. anxiety, severity of depressive symptoms, somatisation) and pharmaceutical problems in both intervention arms of the study will be statistically compared with the patients in the control group.

- The frequency of medical contacts with healthcare services, both psychotherapeutic and in other medical fields will be compared between the intervention arms of the study and the control group. This will also be analysed using secondary data from hospital-IT-systems of various hospitals within the region of Western Pomerania and the patients' statutory health insurances.

- Descriptive analysis of the frequency of patients' crises, the frequency and kind of interventions, initiated by the project nurses. For this evaluation, the contents of the telephone calls (documented in the project documentation system) and the text-messages have to be analysed and categorized.

## Funding

This study is funded by the Ministry of Social Affairs and Health of the Federal State of Mecklenburg-Western Pomerania (Future fund, Telemedicine Programme).

## Competing interests

The authors declare that they have no competing interests.

## Authors' contributions

NvdB, HJG, HJF, and WH participated in the design of the study. HJG participated in the coordination of the patient recruitment. NvdB drafted the manuscript. All authors read and approved the final manuscript.

## Pre-publication history

The pre-publication history for this paper can be accessed here:

http://www.biomedcentral.com/1471-244X/11/30/prepub
